# Stiff-person syndrome manifesting as unilateral extrinsic ocular musculature paresis

**DOI:** 10.31744/einstein_journal/2025AI0881

**Published:** 2025-06-10

**Authors:** Iago Resende Carvalho, Diogo Fernandes dos Santos

**Affiliations:** 1 Universidade Federal de Uberlândia Uberlândia MG Brazil Universidade Federal de Uberlândia, Uberlândia, MG, Brazil.; 2 Universidade Federal de Uberlândia Hospital de Clínicas Department of Neurology Uberlândia MG Brazil Department of Neurology, Hospital de Clínicas, Universidade Federal de Uberlândia, Uberlândia, MG, Brazil.

Stiff-person syndrome (SPS) is an autoimmune disease classically characterized by progressive muscular stiffness and lower back pain with sudden episodes of muscular spasm and, in certain cases, dysautonomia.^(1)^ In general, the disease is diagnosed based on the presence of high anti-glutamic acid decarboxylase (anti-GAD) antibody titers both in the serum and cerebrospinal fluid (CSF), axial muscle stiffness in the special abdominal and thoracolumbar paraspinal fluid leading to hyperlordosis, painful spasms with well-defined triggers, electromyographic evidence of continuous motor unit activity, and the exclusion of alternative diagnoses.^(2)^ In general, the disease evolves with trunk stiffness, followed by progressive limb rigidity and muscle hypertrophy.^(3)^

Although the SPS-related literature is well-established, little evidence is available concerning the ophthalmological manifestations of this disease.^(4,5)^ Recently, certain studies discussed the potential association of GAD with the extrinsic muscular paresis of the eye, leading to ptosis and diplopia.^(6)^

We report the case of a previously healthy 32-year-old woman with a 30-day history of right eyelid ptosis and divergent inferior strabismus of the right eye without diplopia, headaches, visual acuity impairment, or symptom fluctuation. In addition, she reported recurrent low back pain as well as episodes of sudden painful and sustained involuntary muscular contractions along with episodes of tachycardia, diaphoresis, and hyperthermia.

**Figure 1:**
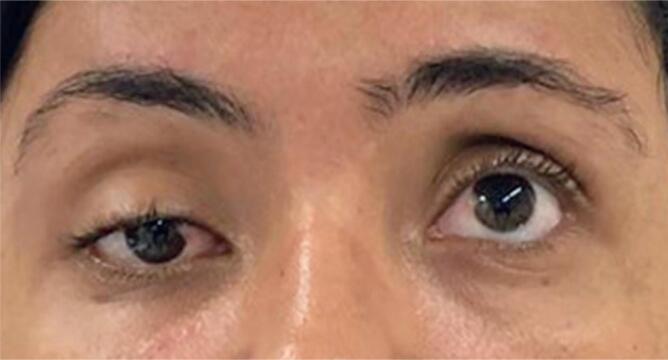
Static eye inspection revealed right eyelid ptosis, eyeball abduction, and internal rotation

Physical examination revealed the eyelid ptosis and eyeball abduction of the right eye as well as internal rotation and slight paresis of the right superior rectus muscle (Figure 1). Moreover, she presented with lumbar hyperlordosis, parasternal, lumbar, and thigh muscular hypertonia as well as symmetric hyperreflexia.

Her magnetic resonance imaging indicated no abnormalities of the neuroaxis or orbit, and her cerebrospinal fluid analysis yielded optimal results. We performed an extensive laboratory evaluation to rule out the possibility of thyroid disorders or systemic infectious and inflammatory diseases. Electroneuromyographic evaluation revealed the presence of continuous muscle contractions, especially in the axial muscles. Repetitive stimulation tests performed on the facial muscles were also optimal.

We measured serum anti-GAD levels using an enzyme immunoassay (reagent >10.0IU/mL), obtaining titers above 200.0IU/mL, thereby confirming the SPS diagnosis.
